# Molecular mode of action of NKP-1339 – a clinically investigated ruthenium-based drug – involves ER- and ROS-related effects in colon carcinoma cell lines

**DOI:** 10.1007/s10637-016-0337-8

**Published:** 2016-03-18

**Authors:** Lea S. Flocke, Robert Trondl, Michael A. Jakupec, Bernhard K. Keppler

**Affiliations:** Institute of Inorganic Chemistry, University of Vienna, Währinger Straße 42, 1090 Vienna, Austria; Research Platform “Translational Cancer Therapy Research”, University of Vienna, Währinger Straße 42, 1090 Vienna, Austria

**Keywords:** Ruthenium, Oxidative stress, ER stress, Unfolded protein response

## Abstract

Sodium *trans*-[tetrachloridobis(1*H*-indazole)ruthenate(III)] (NKP-1339) is a clinically investigated ruthenium-based metal complex, which shows promising results in solid tumors, such as non-small cell lung cancer, colorectal carcinoma, and most distinctively in gastrointestinal neuroendocrine tumors. In previous studies, fast binding to albumin as well as transferrin could be shown. The enhanced permeability and retention (EPR) effect, which is diversely being exploited for tumor targeting, could therefore be applicable for NKP-1339. Here we studied the serum dependence of its biological activity in various methods, influencing its cellular accumulation, cytotoxicity as well as the generation of reactive oxygen species (ROS). ROS lead to Nrf2 activation, which is known to activate antioxidant response gene transcription. GRP78 down-regulation on the protein level suggests ER associated protein degradation (ERAD) as a mode of action, as RNA levels are only mildly affected. Another important part for the mode of action is endoplasmic reticulum (ER) stress, as different factors are highly upregulated on the protein level. For example PERK, a transmembrane receptor which is released by GRP78 when the ER is disturbed, is upregulated and phosphorylated. EIF2α is phosphorylated, which leads to an inhibition of CAP-dependent translation and other stress responses. The transcription factor CHOP (DDIT3), which promotes ER stress dependent apoptosis, is time and concentration dependently upregulated. Finally cytotoxicity tests could prove that inhibition of ER stress and ER stress-mediated apoptosis leads to decreased cytotoxic effects of NKP-1339, which highlights the involvement of this mechanism in the mode of action.

**Electronic supplementary material:**

The online version of this article (doi:10.1007/s10637-016-0337-8) contains supplementary material, which is available to authorized users.

## Introduction

The idea of ruthenium-based cancer chemotherapy is fueled by the facts that some ruthenium compounds accumulate preferably in tumor tissue, ruthenium has, compared to platinum(II), additional coordination sites, and a variety of ruthenium complexes show redox behavior under physiological conditions. As ruthenium-based complexes tend to show lower general toxicities than platinum-based drugs, higher doses can be administered [1, 2]. Three ruthenium-based compounds have been investigated in clinical studies recently, namely NAMI-A, KP1019 and NKP-1339. KP1019 and NKP-1339 bind to transferrin and albumin very fast, whereby adduct formation with albumin is preferred to transferrin [3, 4], and can thereby take advantage of drug delivery by the enhanced permeability and retention (EPR) effect. The EPR effect leads to enhanced accumulation of macromolecules (> 40 kDa), such as albumin, in solid tumors, where they are retained for many hours due to a lack of efficient lymphatic drainage [5]. The serum concentration was shown to have a significant impact on the P-glycoprotein-modulating activity of KP1019 in the leukemia cell line HL60 [6]. In cell culture, binding of KP1019 or NKP-1339 to albumin leads to a decrease in activity, as no EPR effect can be observed and conditions in vitro are therefore probably less favorable for protein-mediated uptake into cancer cells. Once NKP-1339 is delivered to the cell, it can exhibit its cytotoxic activity.

Evidence for clinical anticancer effects of NKP-1339 was already reported from a phase I dose-escalation study where dose proportionality of C_max_ and AUC_0–192_ of the first dosing interval could be observed. Furthermore, patients with gastrointestinal neuroendocrine tumors have experienced partial response or disease stabilization. Stable disease could be observed for a wide variety of tumors, including non-small cell lung cancer (NSCLC), sarcoma and colorectal cancer. The adverse effects are manageable, most commonly mild to moderate nausea, vomiting and fatigue [7].

As enhanced ER stress is a promising strategy against cancer [8], we investigated its role in the mode of action of NKP-1339. The ER is the organelle which is in charge of correct protein maturation, folding and secretion. If too many misfolded proteins are accumulated, the ER starts the unfolded protein response (UPR). If only mild disturbances occur, it enables the organelle by stopping the cell cycle to repair damages, but if a certain threshold is exceeded it can induce apoptosis. It is a mechanism executed via three branches. The three transmembrane receptors PERK, ATF6 and IRE1α are bound by GRP78, which senses and binds damaged proteins, thereby releasing the receptors and starting UPR signaling. The first transmembrane receptor which is released is PERK. Thereupon eIF2α is phosphorylated, which inhibits CAP-dependent translation. ATF4 is CAP-independent and therefore upregulated following eIF2α phosphorylation. The transcription factor ATF4 translocates to the nucleus and induces stress response, expression of genes for amino acid transport and synthesis, and finally CHOP [9]. CHOP is a protein which is involved in the switch to apoptosis and induces Bcl2 downregulation and DR5 (death receptor 5) activation. Activated PERK also leads to a translocation of Nrf2, which is followed by upregulation of genes having an antioxidant response element (ARE) in their promotor, e.g., heme oxygenase 1 (HO-1) and glutathione S-transferase (GST) [10]. When ATF6 is released from GRP78, it is cleaved and translocates to the nucleus. In the nucleus, different chaperones as well as genes for protein degradation are upregulated. In addition, XBP1 is spliced, which is also triggered by release of the third transmembrane receptor IRE1α from GRP78. Splicing of XBP1 mRNA leads to a frameshift and thereupon translation into the functional protein, which, after translocation to the nucleus, activates gene expression of chaperones, as well as genes for protein degradation and p58^IPK^. P58^IPK^ downregulates eIF2α phosphorylation, as well as ATF4 upregulation and CHOP activation via a negative feedback loop [11].

In this study, we reveal the involvement of ER stress in the mode of action of NKP-1339 via Western blotting analysis showing upregulated PERK, p-eIF2α and CHOP. ROS are generated and Nrf2 translocates to the nucleus. Activity is enhanced when serum concentration is reduced, reflecting reduced binding of NKP-1339 to serum proteins, which is considered advantageous only in vitro, though (for reasons explained above).

## Materials and methods

### Reagents and antibodies

NKP-1339 was synthesized as previously reported [12], dissolved in DMSO (40 or 80 mM stocks) and diluted to final concentrations in cell culture medium. MEM, PBS and trypsin were purchased from Sigma-Aldrich (Vienna, Austria). Nrf-2 and 2° FITC coupled antibodies were purchased from Santa Cruz Biotechnology (Dallas, TX, USA). Cycloheximide (CHX) and c-Jun N-terminal kinase (JNK) inhibitor SP600125 were purchased from Abcam (Cambridge, UK). The antibodies against GRP78, p-eIF2α, PERK and β-actin, as well as horseradish peroxidase-labeled anti-mouse igG and anti-rabbit igG were purchased from Cell Signaling Technology (Danvers, MA, USA).

### Cell culture

HCT116 and SW480 (both human colorectal carcinoma) cells were provided by Brigitte Marian (Institute of Cancer Research, Department of Medicine I, Medical University of Vienna, Austria). Cells were grown in Eagle’s minimal essential medium containing 10 % FCS, 1 mM sodium pyruvate, 4 mM l-glutamine and 1 % non-essential amino acids at 37 °C under 5 % CO_2_ and humidified conditions. Cells were passaged twice a week and seeded for experiments in exponential growth phase 24 h before treatment. Cell lines where authenticated and proven to be free of any contamination with mycoplasma by Multiplexion (Heidelberg, Germany).

### Cellular accumulation

120 000 cells/ml were seeded in 2.5 ml complete MEM per well into 6-well plates (CytoOne, Starlab, UK), allowed to settle and recover for 24 h, washed 3 times with 3 ml PBS each and then treated with 100 μM NKP-1339 or medium alone, each containing 2 %, 5 % or 10 % FCS for 2 h. The cells were washed again 3 times with 3 ml PBS. The same treatment was performed to a parallel plate, three replicates for each treatment, from which cells (instead of being lysed by acid treatment) were detached by trypsinization for counting of cell numbers. The samples were treated by addition of 500 μl subboiled HNO_3_. Cell lysis was left for 1 h and then 400 μl of the sample were added to 7.6 ml ddH_2_O and analyzed by ICP-MS as described previously [13]. Three independent biological replicates were performed, each with three technical replicates.

### MTT assay

Cytotoxicity was assessed by the MTT assay. 2000 cells were seeded per well in 100 μl complete MEM into 96-well plates (CytoOne, Starlab, UK), allowed to settle and recover for 24 h, washed twice with PBS, and then 100 μl MEM containing 2 % or 10 % FCS were added either alone or containing CHX or JNK inhibitor. The test compound was dissolved in DMSO, appropriately diluted in MEM and added to the cells in 100 μl per well in triplicates. Incubation with the test compound alone or in combination with the inhibitors took place in the incubator at 37 °C, 5 % CO_2_ under humidified conditions for 96 h. CHX was used in a concentration of 1.25 μM and JNK in 10 μM (those concentrations were found to be the highest not inhibiting cell proliferation alone). Then MEM was replaced with 6× RPMI1640 medium: 1× MTT (3-(4,5-dimethyl-2-thiazolyl)-2,5-diphenyl-2H-tetrazolium bromide) solution in phosphate-buffered saline (5 mg/ml). After 4 h, the RPMI1640/MTT solution was removed and the formed formazan dissolved in 150 µl DMSO per well. Absorption was measured at 550 nm (and 690 nm as a reference wavelength) with a microplate reader (Biotek ELx808). Concentration–effect curves were calculated relative to untreated controls, and IC_50_ (50 % inhibitory concentration) values were interpolated. Results are means ± standard deviations from at least three independent experiments.

### 2′,7′-Dichlorofluorescin diacetate (DCFH-DA) assay

Twenty-five thousand cells were seeded in 100 μl complete MEM per well in a 96-well plate (CytoOne, Starlab, UK). Cells were allowed to settle and recover for 24 h and washed once with Hanks Balanced Salt Solution (HBSS) supplemented with 1 % FCS. The cells were then incubated for 45 min at 37 °C with 25 μM DCFH-DA stock (1 % DMSO in HBSS containing 1 % FCS) and then treated in MEM without phenol red containing 2 %, 5 % or 10 % FCS. 200 μM H_2_O_2_ were used as positive control and pure MEM without phenol red containing 2 %, 5 % or 10 % FCS as negative control. NKP-1339 was dissolved in DMSO to 40 mM, diluted in MEM and added to the cells at final concentrations of 50 μM, 100 μM and 200 μM. Fluorescence was measured over 14 h every 10 min with the multi-mode microplate reader Synergy HT (Biotek) (excitation: 480 nm/emission: 516 nm). Results are presented as treated over control. Two independent biological replicates were performed, each with three technical replicates.

### Immunofluorescence microscopy

Two-hundred thousand cells were seeded on cover slips in a 6-well plate (CytoOne, Starlab, UK) to achieve a 40–70 % confluence 24 h later (on the day of fixation). Liquid was aspired and cells covered for 15 min in 2.5 ml 4 % formaldehyde diluted in 37 °C warm PBS. Cells were washed three times with PBS and 60 min blocked in 1 ml blocking buffer (1× PBS, 5 % BSA, 0.3 % Triton-X™ 100). Then 1° antibody anti-Nrf-2 was added 1:100 in antibody dilution buffer (1× PBS, 1 % BSA, 0.3 % Triton-X™ 100) and incubated over night at 4 °C. The cells were then washed three times, each for 5 min with 2 ml PBS, and then incubated with FITC labeled 2° anti rabbit AB for 2 h at room temperature in the dark. The cells were washed three times, each 5 min with 2 ml PBS, again and 300 nM DAPI in PBS added to the cells for 5 min. Then cells were rinsed several times with PBS and finally mounted in PBS, to be viewed under the fluorescence microscope using the appropriate filters. FITC is excited at 485 nm giving an emission of 514 nm. A BX40 fluorescence microscope with an F-View CCD Camera, Cell^F fluorescence imaging software, and oil immersion objective lens (60× magnification) (all from Olympus, Vienna, Austria) were used. DAPI is excited at 358 nm giving an emission of 561 nm. At least three independent biological replicates were performed, each with about 100 cells analyzed.

### Western blotting

Two-hundred thousand cells were seeded into 6-well plates (CytoOne, Starlab, UK), allowed to settle and recover for 24 h, washed twice with PBS, and then 2 ml MEM containing 2 % or 10 % FCS were added. Then 50 μM, 100 μM or 200 μM NKP-1339 were added for the indicated time periods. 0.5 mM thapsigargin (TG) were used as positive control. Proteins were extracted by lysis with radioimmunoprecipitation assay (RIPA) buffer including 1× protease and 1× phosphatase inhibitor cocktails (Sigma-Aldrich). Per blot, the same amount of protein per lane was electrophoretically separated by size and blotted onto a polyvinylidene fluoride (PVDF) membrane by using a semi-dry blotter (Peqlab, Erlangen, Germany). Then the membrane was blocked for 1 h at room temperature with 5 % BSA in Tris-buffered saline/Tween 20 buffer. The 1° antibody was diluted in 5 % BSA in Tris-buffered saline/Tween 20 buffer according to manufacturer guidelines and incubated at 4 °C over night. Anti-β-actin was used as loading control. The membrane was washed three times with 5 ml PBS and the 2° antibody coupled to horseradish peroxidase was diluted 1: 2000 in 5 % BSA in Tris-buffered saline/Tween 20 buffer and incubated for 1 h at room temperature. Pierce SuperSignal Enhanced chemoluminescent (ECL) substrate (Thermo Fisher Scientific, Inc., Rockford, IL) was incubated for 5 min and detected by a Fusion FX7 chemiluminescence detection system (Vilber Lourmat, Eberhardzell, Germany).

### Reverse transcriptase quantitative PCR (rt qPCR)

High purity RNA was extracted from monolayer cells by using RNeasy Mini Kit (Qiagen, Vienna, Austria) following manufacturer guidelines. cDNA was reversely transcribed by using High-Capacity Reverse Transcription Kit (Applied Biosystems, Bleiswijk, Netherlands) following manufacturer guidelines. Rt qPCR was performed by using a Rotor Gene Q instrument (Qiagen, Vienna, Austria). For Eva Green-based amplification assessment 10 ng template/rxn were amplified with 10 μM forward and reverse primer and 1× HOT FIRE Pol® EvaGreen® qPCR Mix Plus (Medibena, Vienna, Austria). For probe-based amplification assessment (for XBP1 spliced) 10 ng template/rxn were amplified with 10 μM forward and reverse primer, 10 μM spliced and unspliced probe and 1× HOT FIRE Pol® Probe qPCR Mix Plus (ROX). All genes were normalized to the reference gene Oaz. The temperature protocol initiated a hot start: 15 min, 95 °C. Then 40 cycles of 60 s 60 °C, 30 s 95 °C. The high-resolution melting curve was analyzed to ensure that the amplified region was the one of interest. The primer sequences as well as the hydrolysis probes were designed by using Primer Express software (version 2.0; Applied Biosystems, Vienna, Austria). Specificity was investigated by Primer-BLAST (NCBI, NIH). A series of 10-fold dilutions of a control cDNA from SW480 or HCT116 cells amplified in duplicates was used to generate a standard curve. The amplification efficiencies were calculated from the slope of the dilution row and were at least 88 %. The *n*-fold relative amplification from treated to untreated was calculated. At least three independent biological replicates were performed, each with two technical replicates.

### Statistical analysis

Results were analyzed by one-way ANOVA by using GraphPad Prism (version 5.0; GraphPad Software, Inc., La Jolla, CA). The significance level for the test is 5 %.

## Results

**Fig. 1:**
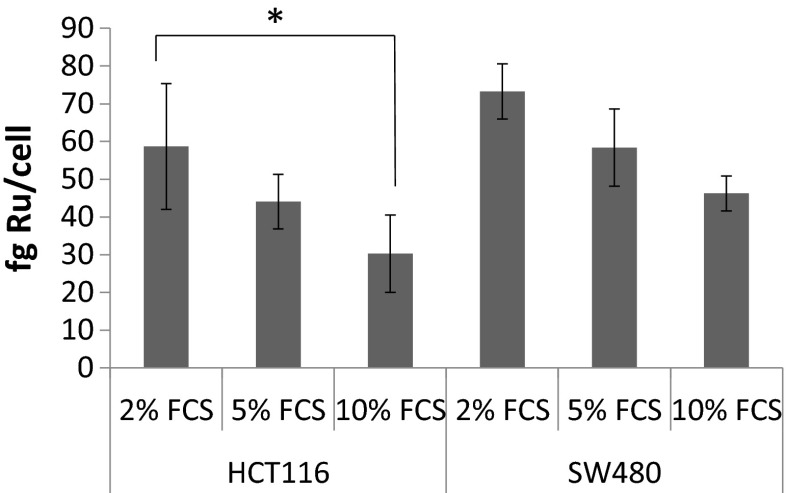
Influence of serum content on cellular accumulation of Ru (from NKP-1339) in the two colon carcinoma cell lines HCT116 and SW480 (*n* = 3). Cells were treated for 2 h with 100 μM NKP-1339 in media containing different serum concentrations (2 %, 5 % or 10 % as indicated). An inverse correlation between cellular accumulation and serum content could be observed

In this study, we could show by ICP-MS measurements that the extent to which NKP-1339 is taken up into the cell within 2 h strongly depends on the serum content (2 %, 5 % or 10 %) of the medium. In both colon carcinoma cell lines, ruthenium accumulation is negatively correlated to the serum concentration (Fig. 1). One-way ANOVA proves a significant difference between 2 % FCS and 10 % FCS in HCT116 cells. Overall, SW480 cells show a higher cellular accumulation and slightly lower serum dependency (with a discernible trend, but not reaching the significance level) than HCT116 cells.

**Fig. 2:**
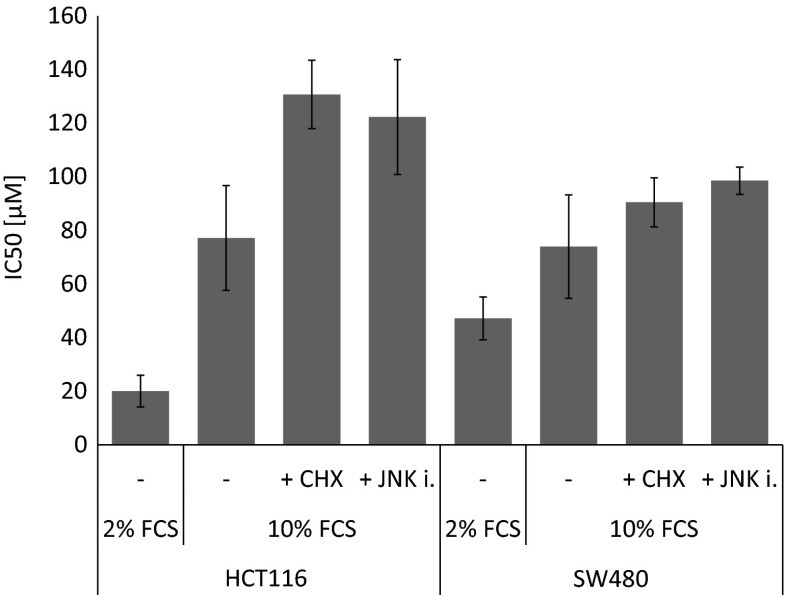
Cytotoxicity of NKP-1339 in the two colon carcinoma cell lines HCT116 and SW480 treated in medium containing 2 % or 10 % FCS and co-treatment with inhibitors of ER stress or responses thereto (*n* = 3). Cytotoxicity is illustrated by the half maximal inhibitory concentration (IC_50_). Cytotoxicity is increased when serum concentration is reduced in both cell lines. Inhibiting protein translation by CHX as well as inhibiting translation from ER stress to apoptosis by the JNK inhibitor SP600125 decreases cytotoxicity

SW480 and HCT116 are both epithelial, adherent cell lines from the same histological background (colon carcinoma) but are known to show different chemoresistance profiles. To study the influence of serum concentration on the cytotoxic properties of the ruthenium complex, MTT assays were performed, and pronounced differences in IC_50_ values could be observed. In good accordance with the cellular accumulation experiments, NKP-1339 exerts higher cytotoxicity if less of serum is present (Fig. 2). The IC_50_ value is four times increased in HCT116 cells if the serum concentration is raised from 2 % to 10 %. Compared to this cell line, the cytotoxicity in SW480 cells is only mildly reduced by increased serum content. Remarkably, the sensitivity to NKP-1339 at low serum concentration is lower in the P-glycoprotein (P-gp) overexpressing cell line SW480 despite a tendency for slightly higher cellular ruthenium accumulation. This might indicate the presence of some kind of intrinsic resistance independently of P-gp.

**Fig. 3:**
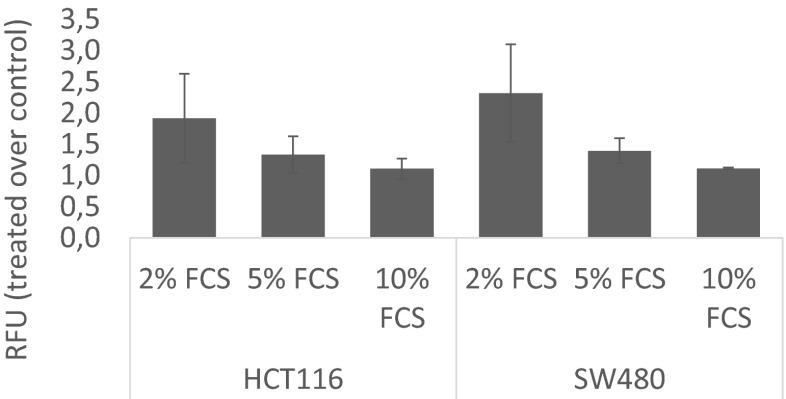
Generation of reactive oxygen species (ROS) in HCT116 and SW480 cells after 1 h treatment with 200 μM NKP-1339 (*n* = 2) in the presence of different serum concentrations. The relative fluorescence units plotted on the y-axis indicate ROS levels, which are inversely dependent on serum content of the medium

The DCFH-DH stained cells showed that NKP-1339 induces elevated levels of ROS. The level of induced ROS is inversely correlated to the serum concentration. This correlation again stresses the importance of serum content for the effectivity of the ruthenium compound. We quantified the levels of ROS over 14 h and chose 1 h as the most distinctive time point to show that ROS levels in both cell lines are comparable, and both consistently show an inverse relationship between ROS formation and serum concentration (Fig. 3). Results for a 2 h time course are shown in Fig. S1 (supplementary data).

**Fig. 4:**
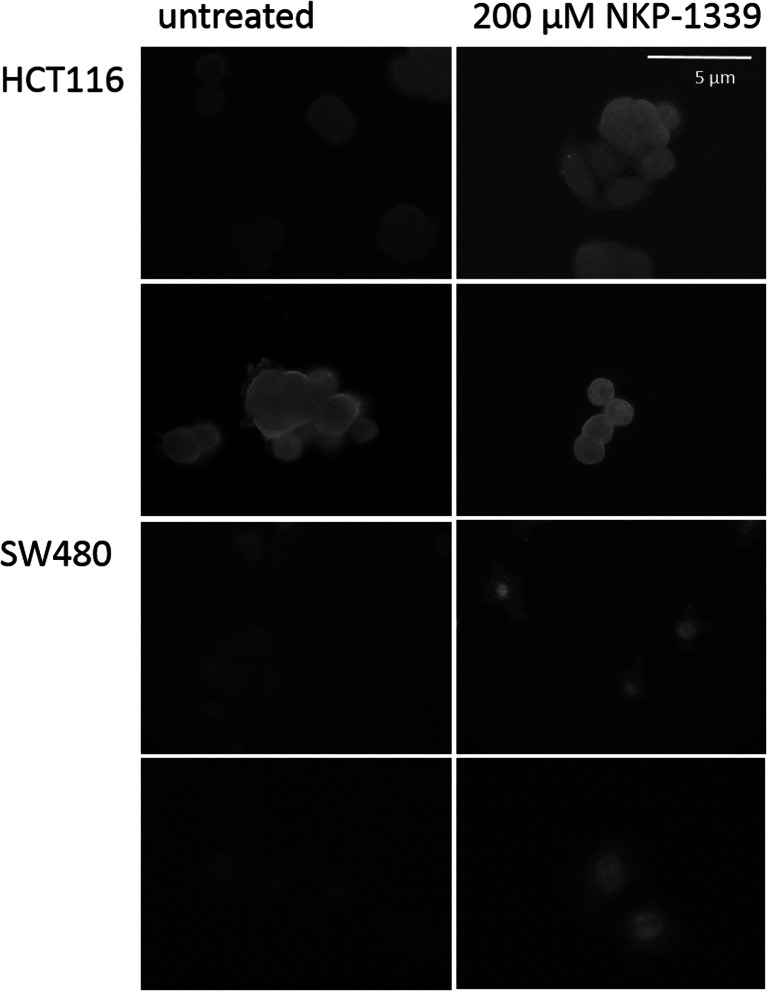
Nrf2 translocation. Cells treated for 6 h in medium containing 2 % FCS. Upon treatment with NKP-1339, Nrf2 is translocating to the nucleus. Pictures depict typical results. Scale bar applies to all images

The well-known transcription factor Nrf2 was found to be translocating to the nucleus upon treatment with NKP-1339. In Fig. 4 translocation is shown after 6 h hours with 2 % serum concentration in the medium. Overall, translocation seems to be more pronounced in SW480 than in HCT116 cells. In the nucleus, Nrf2 is known to activate genes that contain antioxidant response elements (ARE) in their promoter region. Those ARE genes induce a detoxifying cell answer.

In this study, we could show that three important key factors of ER stress are highly upregulated on the protein level upon 24 h exposure to NKP-1339. The transmembrane receptor protein kinase R (PKR)-like endoplasmic reticulum kinase (PERK), which is encoded by the gene EIF2AK3, is phosphorylated as the band is shifted and vanishes at very high concentrations of NKP-1339. This effect is less pronounced in SW480 cells, where the phosphorylation is only visible in cells treated in medium containing 10 % serum. The phosphorylation upon NKP-1339 treatment at up to 100 μM leads to an activation of the kinase. The degradation at higher concentrations of NKP-1339 might be caused by ERAD as will be discussed below. The 78 kDa glucose-regulated protein (GRP78, also BiP or HSPA4) – a chaperone which senses damaged proteins by binding hydrophobic patches [14] – is upregulated when cells are treated with low concentrations of NKP-1339, but when 100 μM are applied in media containing 10 % FCS or when 200 μM are applied in cells treated in media containing 2 % FSC the protein approaches the basal level again. In SW480 cells degradation of GRP78 after treatment with 200 μM NKP-1339 is observed. PeIF2α is upregulated, but this effect becomes less distinct at high concentrations of NKP-1339. CHOP, which is responsible for the switch from the pro-survival mode of UPR to proapoptotic signaling, is overexpressed in both cell lines, HCT116 and SW480. In HCT116 cells, this transcription factor shows a pronounced activation when cells were treated in medium containing 2 % FCS but not when in medium containing 10 % FCS.

To further investigate the role of ER stress in the cellular effects of NKP-1339, different key factors were analyzed on the mRNA level at various time points from 1 h to 48 h. To illustrate effects on the mRNA level in Fig. 6, data from 4 h exposure experiments are displayed, which is shorter than that used in Western blotting experiments (24 h) which showed pronounced changes in expression of certain factors. CHOP mRNA expression is upregulated in both cell lines when cells were grown in media containing 2 % or 10 % FCS. Interestingly, the effects in HCT116 cells are more pronounced when the serum concentration is 10 %. Similar results were obtained for spliced XBP1 which via a frameshift during splicing leads to expression of the functional protein. In HCT116 cells, the XBP1 splicing effect is more pronounced in cells treated in medium containing 2 % FCS, whereas in SW480 cells again the more pronounced effect is observed in medium containing 10 % FCS. The other factors: ATF4, IRE1α as well as GRP78 show no pronounced alterations on the mRNA level at any incubation time.

**Fig. 5:**
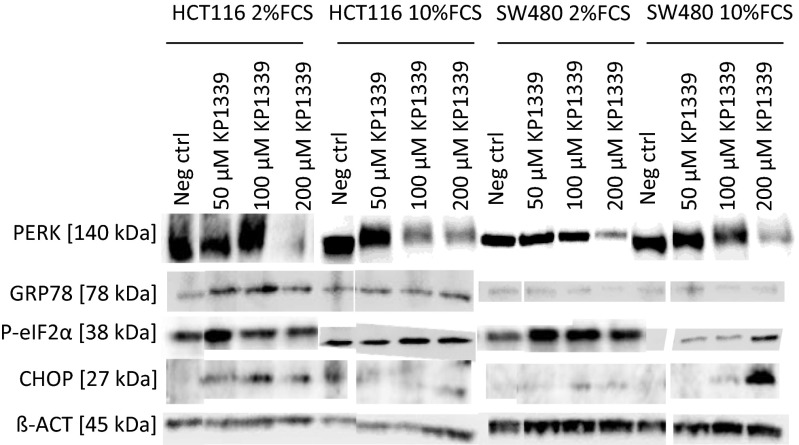
Western blot analysis showing p-eIF2y α and CHOP upregulation, PERK phosphorylation as well as Grp78 regulation (*n* = 3). Incubation time is 24 h. ß-Actin was used as a loading control

**Fig. 6:**
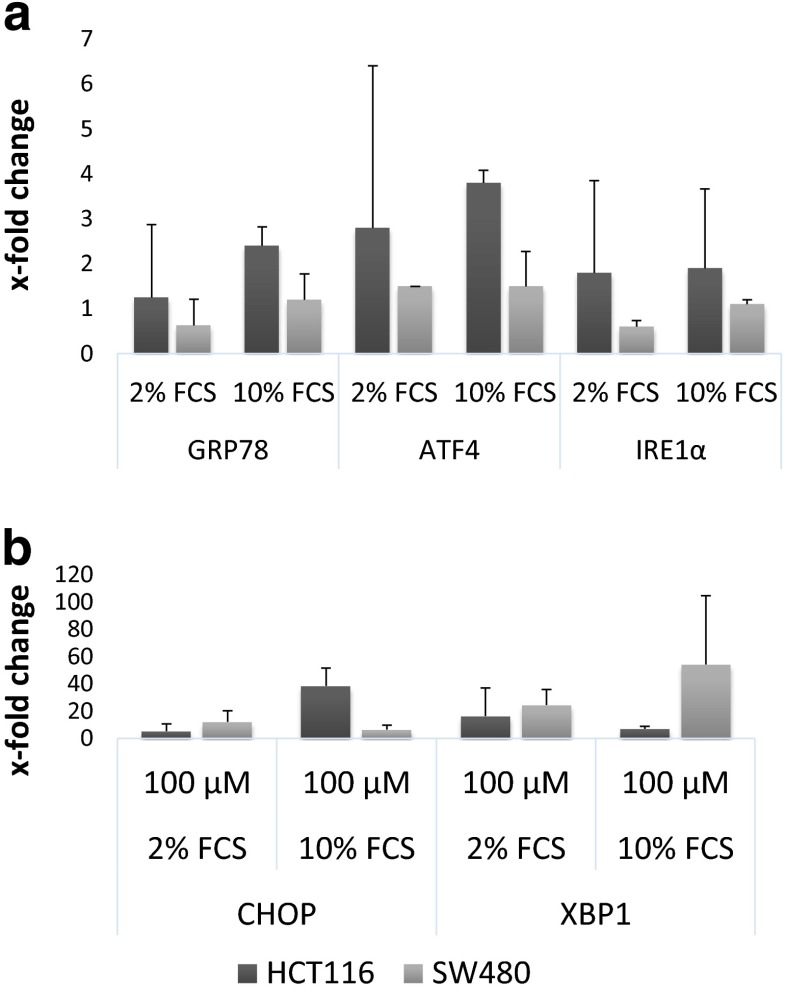
Rt qPCR of UPR key factors in two cell lines upon 4 h exposure to NKP-1339 in media containing 2 % or 10 % FCS (*n* = 3). GRP78, ATF4 and IRE1α show no major upregulation on the mRNA level (**a**). CHOP and XBP1 are slightly or even distinctly upregulated. CHOP shows the highest upregulation in HCT116 cells treated in medium containing 10 % FCS, and XBP1 in SW480 cells treated in medium containing 10 % FCS (**b**). Note the differently scaled x-axes

GRP78 was found to be downregulated on the protein level in SW480 cells treated with 200 μM NKP-1339 in medium containing 10 % FCS (Fig. 5), whereas for GRP78 no effect on mRNA expression was found, which suggests that ER-associated protein degradation (ERAD) is involved. ERAD is known to be induced by ER stress [15] to reduce the protein burden for the organelle, and as NKP-1339 induces ER stress ERAD seems plausible (Fig. 6).

Finally, the relevance of ER stress for the mode of action of NKP-1339 was investigated by combined application of NKP-1339 with specific inhibitors of ER stress or cellular responses thereto. CHX is produced by the bacterium *Streptomyces griseus* and an inhibitor of eukaryotic protein synthesis by preventing translational elongation. This leads to a reduced protein load for the ER, which can relieve ER stress. The c-Jun N-terminal kinase (JNK) inhibitor SP600125 inhibits c-Jun N-terminal kinase ATP-competitively, which prevents ER stress-induced apoptosis. Both inhibitors are able to slightly decrease the cytotoxic properties of NKP-1339 in both colon carcinoma cell lines, with IC_50_ values increasing by 21 % for CHX in HCT116 cells and by 70 % for CHX in SW480 cells. The JNK inhibitor SP600125 leads to increases of IC_50_ values by 33 % in HCT116 cells and 58 % in SW480 cells (Fig. 2).

## Discussion

This study gives valuable insights into the mode of action of the clinically investigated ruthenium complex NKP-1339 in the two colon carcinoma cell lines HCT116 and SW480. Colon carcinomas are highly mutated tissues and can be characterized by mutations causing resistance, such as in p53, and phenotypic changes such as upregulation of Bcl2 and multidrug resistance (MDR) genes. It was shown previously that NKP-1339 reacts fast with the protein albumin [16], which is the most abundant protein in human serum with a concentration of about 600 μM [17] and contained in FCS used for cell culture as well. Albumin is accumulated in tumor tissue based on the EPR effect, which, however, is not reflected in cell culture settings. As a result, binding to albumin, though desirable for the tumor selectivity it may mediate in vivo, seems disadvantageous in vitro as it leads to decreased activity of the compound. The EPR effect is, however, only one of the components contributing to enhanced albumin uptake into tumor tissue. The second major albumin uptake mechanism is the gp60/SPARC-activated pathway (gp60 is a 60-kDa endothelial cell membrane albumin-binding protein localized in caveolae; SPARC stands for secreted protein, acidic and rich in cysteine) as well as hyperactive caveolae transport. In patients with advanced solid tumors, a third pathway can be activated in association with hypoalbuminemia (decreased serum albumin level) [18]. Cellular accumulation studies revealed an inverse correlation between serum content and cellular accumulation, which explains why the compound shows a lower cytotoxic potency when serum content is increased to the usual value. Altogether this clarifies why NKP-1339 is poorly active in the cell culture setting in contrast to its therapeutic efficacy (with mild side effects) in patients with solid tumors in clinical studies [7]. Accumulation and long retention in tumor tissue may compensate for the drop in activity initially elicited by serum protein binding. In vitro, however, high drug concentrations are required to be able to detect appreciable activity, since serum proteins tend to obscure its cellular effects.

Effects of serum proteins on the biological activity have occasionally been reported in the literature also for other anticancer ruthenium complexes. The consequences of albumin binding for analogs of NKP-1339 with substituted pyridine ligands were shown to be divergent, depending on their tendency for hydrophobic versus coordinate protein interactions [19]. In the cell culture setting, the more easily reversible non-coordinate interactions are favorable for cytotoxicity, whereas coordinate binding tends to decrease it, in line with our results. For the clinically developed anticancer Ru(III) compound NAMI-A, decreased effects on cell cycle progression as well as cell viability upon adduct formation with albumin or transferrin were shown as well [20]. The re-adhesion rate (as an aspect of the metastatic process inhibited by NAMI-A) of cancer cells was reported to be only slightly (though significantly) less reduced when NAMI-A is applied in albumin-bound form in vitro, suggesting that biological activity is basically maintained [21].

NKP-1339 was also shown to induce elevated levels of reactive oxygen species in an inversely serum-dependent manner, which further stresses the influence of serum proteins. Both cell lines show similar ROS levels, but IC_50_ levels in SW480 cells are higher (at least for 2 % FCS); this implies that the cell line HCT116 is more sensitive towards ROS than SW480. For KP1019 (the indazolium salt analog of NKP-1339), it was shown in previous studies that cytotoxicity is reduced by *N*-acetylcysteine (NAC) addition, suggesting an important role for ROS in the mode of action [22]. ROS are known to induce Nrf2 translocation from the cytoplasm into the nucleus. In the nucleus, this transcription factor induces different genes containing an ARE in their promotor site. We could show that translocation of Nrf2 upon NKP-1339 exposure indeed occurs in colon cancer cell lines. Further ROS lead to protein damage, which leads to an accumulation of misfolded proteins in the ER. Because of enhanced and fast metabolism, cancer cells show an increased level of oxidative stress and ER stress [8]. After exceeding a certain threshold, UPR signaling indicates ER stress, which we could confirm on the protein level. GRP78 as a major ER resident chaperone is one of the key sensors of protein damage, a key regulator of ER stress and responsible for processing of misfolded proteins. It has been proposed as a major target for NKP-1339 (Dickson et al., 2011). Furthermore, GRP78 is correlated with poor patient survival, high pathological grade and relapse in breast, liver, prostate and gastric cancer, as well as colon carcinoma; therefore, it could be applied as a biomarker. The chaperone inhibits activation of caspase 7, cytochrome C release as well as of the BH3-only proapoptotic proteins Bik and Bax [23]. We could find that the chaperone is regulated on the protein level but only mildly influenced on the mRNA level, which suggests a certain involvement of ERAD in the mode of action of NKP-1339. The relevance of ER stress for the cytotoxic properties of the ruthenium compound was confirmed by showing that IC_50_ values are significantly increased when ER stress is inhibited by CHX as well as when ER stress-induced apoptosis is inhibited by a JNK inhibitor.

## Electronic Supplementary Material

ESM 1 (DOCX 77 kb)
