# Supplementary material for: Molecular mode of action of NKP-1339 – a clinically investigated ruthenium-based drug – involves ER- and ROS-related effects in colon carcinoma cell lines
Source: Invest New Drugs. 2016 Mar 18;34:261–8. doi: 10.1007/s10637-016-0337-8 (PMC4859864; doi:10.1007/s10637-016-0337-8)
Supplement: Supplementary file 1 — (DOCX 77 kb) [file 10637_2016_337_MOESM1_ESM.docx]

**Molecular mode of action of NKP-1339
 – a clinically investigated ruthenium-based drug – involves ER- and ROS-related effects in colon carcinoma cell lines**

L. S. Flocke, R. Trondl, M. A. Jakupec, B. K. Keppler

**Supplementary data**

**Table S1. Influence of serum content on the cellular accumulation of Ru (from NKP-1339) in the colon carcinoma cell lines HCT116 and SW480 (n = 3).** Cells were treated as described under Fig. 1.

| Cell line and serum conc. [%] | fg Ru/Cell | Stdev |
| --- | --- | --- |
| HCT116 2% FCS | 58.655445 | 16.66363 |
| HCT116 5% FCS | 44.087876 | 7.256256 |
| HCT116 10% FCS | 30.264562 | 10.23835 |
| SW480 2% FCS | 73.227655 | 7.256256 |
| SW480 5% FCS | 58.347308 | 10.2484 |
| SW480 10% FCS | 46.247488 | 4.644271 |

**Table S2. Cytotoxicity of NKP-1339 in the colon carcinoma cell lines HCT116 and SW480 treated as described under Fig. 2 (n = 3).** Cytotoxicity is illustrated by the half maximal inhibitory concentration (IC_50_). Cytotoxicity is increased when serum concentration is reduced in both cell lines. Inhibiting protein translation by CHX as well as inhibiting translation from ER stress to apoptosis by the JNK inhibitor SP600125 decreases cytotoxicity.

| Cell line | SW480 | | | | **HCT116** | | | |
| --- | --- | --- | --- | --- | --- | --- | --- | --- |
| FCS conc. | 2% | 10% | | | 2% | 10% | | |
| Inhibitor | - | - | CHX | JNK i. | - | - | CHX | JNK i. |
| IC50 | 47 ± 8.0 | 74 ± 19 | 90 ± 9.2 | 99 ± 5.0 | 20 ± 5.9 | 77± 20 | 131 ± 13 | 122 ± 21 |

**
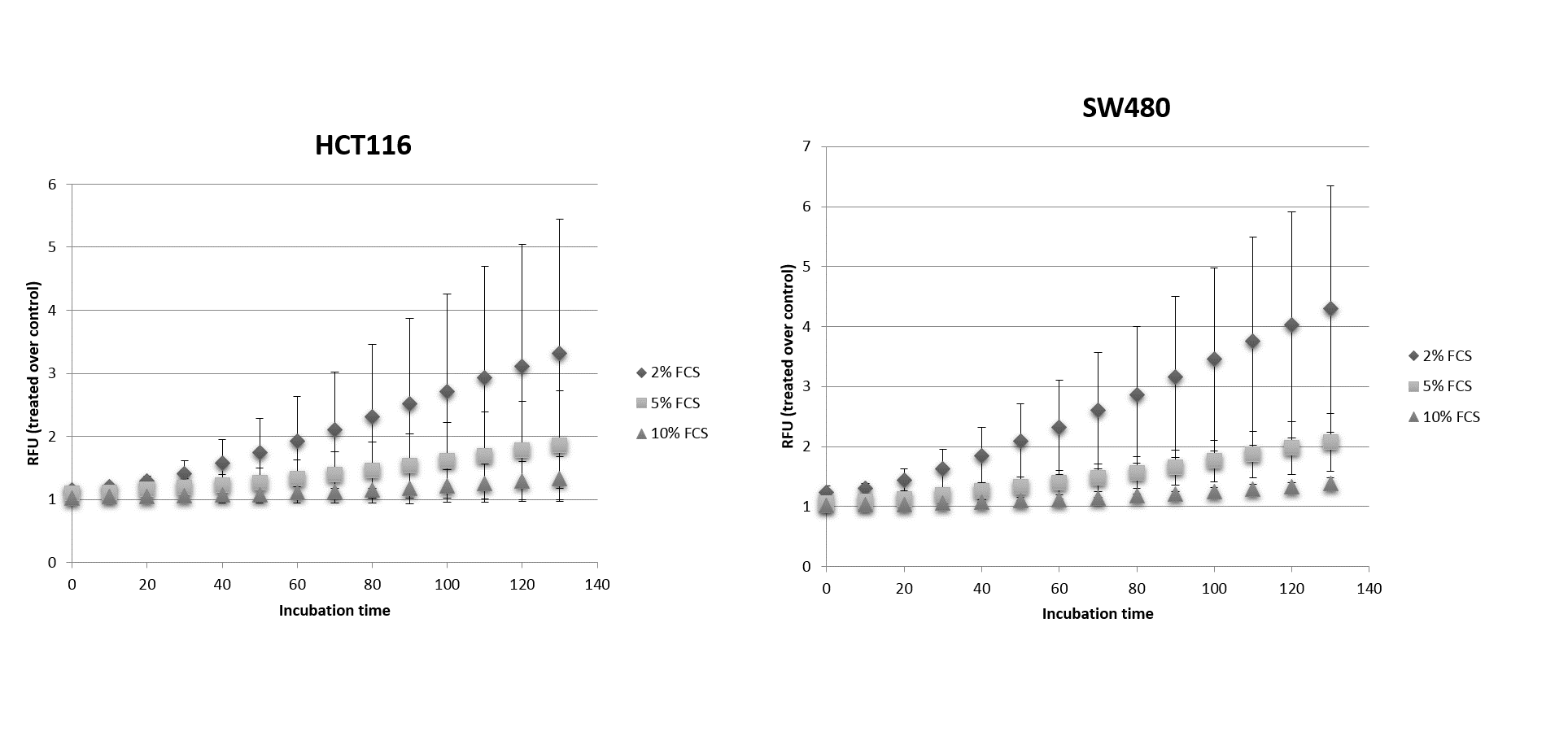
**

**Fig. S1. ROS induction in two colon carcinoma cell lines over 2 h.** Elevation of ROS levels is inversely correlated with serum concentration.
